# Light Sensing beyond Vision: Focusing on a Possible Role for the FICZ/AhR Complex in Skin Optotransduction †

**DOI:** 10.3390/cells13131082

**Published:** 2024-06-22

**Authors:** Tiziana Guarnieri

**Affiliations:** 1 Cell Physiology Laboratory, Department of Biological, Geological, and Environmental Sciences, Alma Mater Studiorum Università di Bologna, Via Francesco Selmi 3, 40126 Bologna, Italy; tiziana.guarnieri@unibo.it; 2 Interdepartmental Center for Industrial Research in Life Sciences and Technologies, University of Bologna, 40126 Bologna, Italy; 3 Consiglio Nazionale delle Ricerche, Istituto per le Applicazioni del Calcolo “Mauro Picone”, Via dei Taurini 19, 00185 Roma, Italy

**Keywords:** skin, light, ultraviolet radiation, chromophores, photosensitizer, aryl hydrocarbon receptor (AhR), 6-formylindolo[3,2-b]carbazole (FICZ)

## Abstract

Although our skin is not the primary visual organ in humans, it acts as a light sensor, playing a significant role in maintaining our health and overall well-being. Thanks to the presence of a complex and sophisticated optotransduction system, the skin interacts with the visible part of the electromagnetic spectrum and with ultraviolet (UV) radiation. Following a brief overview describing the main photosensitive molecules that detect specific electromagnetic radiation and their associated cell pathways, we analyze their impact on physiological functions such as melanogenesis, immune response, circadian rhythms, and mood regulation. In this paper, we focus on 6-formylindolo[3,2-b]carbazole (FICZ), a photo oxidation derivative of the essential amino acid tryptophan (Trp). This molecule is the best endogenous agonist of the Aryl hydrocarbon Receptor (AhR), an evolutionarily conserved transcription factor, traditionally recognized as a signal transducer of both exogenous and endogenous chemical signals. Increasing evidence indicates that AhR is also involved in light sensing within the skin, primarily due to its ligand FICZ, which acts as both a chromophore and a photosensitizer. The biochemical reactions triggered by their interaction impact diverse functions and convey crucial data to our body, thus adding a piece to the complex puzzle of pathways that allow us to decode and elaborate environmental stimuli.

## 1. Introduction

The skin, the largest organ in the human body, has a dual embryologic origin. The epithelial tissue, known as the epidermis, is its outermost layer and originates from the ectoderm. Conversely, the underlying connective tissue, the dermis, arises from the mesoderm. The skin functions as a dynamic barrier, adjusting to changes in the external environment, preserving internal homeostasis, and protecting our body from external threats. [1]. It fulfills its vital role as an interface between the external and internal environments, thanks to the presence of numerous sensors for both chemical and physical stimuli distributed throughout its layers [2]. Among these sensors, the most recognized are found in the olfactory (smell) and gustatory (taste) organs, which enable the interpretation of chemical stimuli in the form of gases and liquids, respectively. Our ability to perceive various physical stimuli in the environment is implemented by a diverse array of skin receptors encompassing touch, temperature, pain, pressure, vibration, as well as more subtle sensations such as tickling and itching. Phototransducers are rarely included in this list, even if melanogenesis, that is, the synthesis of melanins pigments in melanocytes, occurs following skin exposure to UV radiation. This obviously implies the presence of a skin phototransduction system. In mammals, including humans, this process serves a photoprotective function. Throughout the day, the Earth’s surface and its living organisms are perpetually exposed to solar radiation. This radiation consists of ultraviolet (UV) rays with a wavelength between 200 and 400 nm, visible light ranging from 400 to 750 nm, and infrared (IR) radiation spanning from 750 to 2000 nm. Given that, the presence in the skin of transducers stimulated by radiation from this segment of the electromagnetic spectrum can be hypothesized [3]. These sensors make the skin able to perceive changes in their intensity and respond accordingly. This phenomenon plays a crucial role in aligning many physiologic events with the daily patterns of light and darkness, improving behavioral and physiological adaptations to light exposure.

## 2. Visual and Non-Visual Receptors

In Vertebrates, the most commonly known visible light receptors are rods and cones in the retina, the light-sensitive layer of the eye. Rods are specialized for detecting light in low-light conditions (dim light); they play a crucial role in peripheral vision and motion detection, but they do not contribute to color vision. Conversely, cones are specialized for perceiving light in bright conditions and enable our ability to see colors. Both rods and cones contain 11-cis-retinal, a light-sensitive chromophore, derived from vitamin A, which is bound to an opsin (OPN), a G-protein-coupled receptor (GPCR). When a photon of light is absorbed, 11-cis-retinal undergoes isomerization to all-trans-retinal. This geometrical change in configuration leads to a conformational change in the OPN and triggers the activation of the phototransduction cascade. Interestingly, about 0.5–5% of retinal gangliar cells, called intrinsically photosensitive retinal ganglion cells (ipRGCs), are photosensitive but not involved in visual stimuli transmission to the brain. They express OPN4, also called melanopsin, which is most sensitive to short wavelengths (blue) and primarily involved in the detection of ambient light intensity. Consequently, ipRGCs transmit information about the environmental light–dark cycle to the brain’s circadian clock. This clock is responsible for regulating the circadian rhythm, the pupillary light reflex and other processes dependent on the perception of light–dark alternation, including sleep and mood [4]. Intriguingly, OPNs are also present in the skin. The most well studied is OPN4, which is expressed in epidermal keratinocytes, melanocytes, and dermal fibroblasts [5] (Table 1). Cutaneous melanopsin serves as a detector for temperature and ultraviolet A (UVA) radiation but is also a regulator of the melanocytes cell cycle and proliferation. Interestingly, the OPN4 corresponding gene behaves as an oncogene in melanoma [6].

## 3. Melanogenesis: Beyond Its Protective Role

Until now, melanogenesis has been commonly viewed as a reactive response to cellular damage caused by exposure to ultraviolet radiation. [13]. Ultraviolet (UV) rays are a well-known risk factor for inducing skin redness, pain, swelling, and, ultimately, sunburns. The severity of these symptoms depends on the individual’s skin type and the extent of exposure, which, if prolonged and excessive, can damage the DNA in keratinocytes, potentially leading to the development of cancer. To protect the DNA, an internal mechanism known as tanning is initiated. As anticipated, it involves an increase in the production of melanins, which are pigments produced by melanocytes in the basal layer of the epidermis. Melanins are stored in cytoplasmic organelles within melanocytes, the melanosomes, which transfer melanins to adjacent keratinocytes through exocytosis. In this way, they protect the genetic material from the harmful effects of UV light. The process of melanogenesis starts in keratinocytes. Following UV-induced DNA damage, such as the formation of cyclobutane pyrimidine dimer (CPD) and pyrimidine photoproducts [6,13], TP53, a protein responsible for the overall damage response, is activated and triggers the transcription of the precursor protein Proopiomelanocortin (POMC). POMC is then cleaved into smaller peptides, including alpha-Melanocyte-Stimulating Hormone (α-MSH). α-MSH is then released from keratinocytes into the extracellular space and binds to its receptor, the Melanocortin-1 Receptor (MC1R) on the membrane of melanocytes. This G-protein coupled receptor mediates the effects of αMSH on melanogenesis, cell proliferation, and apoptosis. This binding triggers a cascade of reactions that ultimately leads to the production of melanins [14] and their storage in melanosomes.

Melanins production and the transfer of melanosomes are also stimulated by interleukin-1 (IL1) and tumor necrosis factor-alpha (TNFα), which are produced and released in the extracellular space by keratinocytes exposed to UV radiation. Notably, they are two of the most important proinflammatory cytokines, which, in this case, participate in the inflammatory response of the skin to UV exposition. When IL-1 and TNF-alpha bind to their receptors on melanocytes, they activate various intracellular pathways, including the cyclic Adenosine Mono Phosphate (cAMP) and the Mitogen-Activated Protein Kinase (MAPK) ones. These pathways ultimately result in the upregulation of genes involved in melanins production and melanosomes transfer. This simplified overview of melanogenesis highlights that UV rays act as a physical stimulus that induces DNA damage and triggers a series of reactions, including inflammation, aimed at producing molecules that protect against photodamage and regulate thermic responses. These reactions involve a dynamic interplay between neighboring cell types, namely, keratinocytes and melanocytes [15,16].

## 4. Photo (Light)-Sensitive Molecules, Chromophores, and Photosensitizers

Recent studies have identified sensing molecules in the skin that detect various types of radiation within the electromagnetic spectrum. This discovery has led to an expansion of the understanding of how the skin perceives sunlight, as recently discussed by Monteiro de Assis and colleagues [7]. So, photoreception, which is the process starting in retinal photoreceptors that convert light energy into an electrical signal which is transmitted to the brain, can be integrated into optotransduction processes occurring in various body regions, particularly the skin. Optotransduction refers to the process converting electromagnetic waves in chemical and/or electrical signals in biological systems [17]. An important aspect relates to the receptors present in our skin that sense electromagnetic radiation, including visible light. In this context, chromophores and photosensitizers are crucial, serving as central components in light-sensitive processes spanning biology, photochemistry, and phototherapy. Chromophores, such as pigments found in the skin and eyes, primarily contribute to color perception. They possess a conjugated system of double bonds or other electron-rich structures, allowing them to absorb specific wavelengths of light and reflect others, thus creating the perception of color. Examples of chromophores include the benzene ring, the carbonyl group, and the nitro groups [18]. Chromophores can play a role in photobiomodulation (PBM) therapy by absorbing the light of specific wavelengths and converting its physical energy into chemical energy. This energy can then be used to stimulate cellular metabolism and facilitate healing processes [19]. Additional chromophores such as melanins and haemoglobin found in the skin, muscles, and blood vessels can facilitate skin rejuvenation and enhance metabolism when adequately activated. In certain inflammatory conditions, mitochondrial cytochromes, including cytochrome c oxidase (CCO), act as chromophores when exposed to specific wavelengths of light. This stimulation leads to increased ATP production and cellular metabolism, resulting in reduced inflammation and enhanced tissue repair [20].

Conversely, photosensitizers absorb photon energy and transfer it to other molecules or substrates via reactive species produced from photochemical reactions. While chromophores are generally stable and do not produce reactive oxygen species (ROS), photosensitizers are specifically designed to generate ROS and cause damage to nearby molecules. Interestingly, certain endogenous chromophores can exhibit a photosensitizer-like behavior when exposed to UVA radiation, which, compared to UVB and UVC, possesses higher energy and penetrates deeper into the skin layers. As explained earlier, the absorbed energy can either be dissipated as heat or transferred to other molecular species, including oxygen (O_2_), thus leading to the generation of O_2_ radicals and ROS [21]. Both oxygen radicals and ROS exhibit high reactivity, potentially leading to oxidative harm to biomolecules such as lipids, proteins, and DNA. These characteristics have practical applications in photodynamic therapy (PDT) for treating various illnesses. In PDT, a photosensitizer is administered to the patient, and the targeted tissue is then exposed to directed light. The photosensitizer absorbs this light, transferring energy to other molecules and generating ROS. Consequently, nearby cells, including cancerous ones, are damaged [22,23].

To date, a number of endogenous light-sensing molecules, chromophores and photosensitizers have been identified in the skin by various researchers [7,9] (Table 1). These include flavins, such as Flavin Adenine Dinucleotide (FAD) and its reduced forms FADH and FADH2; coenzymes like Nicotinamide Adenine Dinucleotide (NAD), Nicotinamide Adenine Dinucleotide Phosphate (NADP), and their reduced forms NADH, NADH2, NADPH, and NADPH2; carotenoids, fat-soluble organic pigments that absorb specific wavelengths of light, resulting in various shades ranging from yellow to red; melanins and lipofuscin (age pigment); and urocanic acid (P), which acts as a UVB photo protectant in the stratum corneum and plays a role in UV-induced immunosuppression [7,10,11].

In recent years, the role of OPNs has gained increasing recognition in optotransduction [24]. These membrane proteins were initially studied for their role in visual signal transduction in the retina, due to their involvement in light sensing. As anticipated, OPNs are photoreceptor proteins that contain a chromophore, such as a retinal, and function as light-sensitive molecules. Upon the absorption of light, the chromophore within the OPN protein undergoes a change in the configurational isomerism. This triggers a series of biochemical events that ultimately lead to the generation of an electrical signal, such as in the case of visual photoreception, or to a chemical signal, as in non-visual optoreception. Downstream of OPNs activation, several cellular pathways are involved in the signal transduction and response. These pathways [25] include

- GPCR Signaling: OPNs are a type of GPCR. Thus, their activation triggers the G-protein signaling cascade. When the light activates OPNs, a conformational change in the receptor occurs, leading to the exchange of guanosine diphosphate (GDP) for guanosine triphosphate (GTP) on the G-protein alpha subunit. This activates the G-protein, leading to the modulation of various downstream effectors such as adenylyl cyclase, phospholipase C, and ion channels.
- Second Messenger Systems: The activation of OPNs can lead to the production of second messengers such as cyclic guanosine monophosphate (cGMP) and inositol trisphosphate (IP3), which play crucial roles in further signal transduction within the cell.
- Ion Channel Modulation: In retinal photoreceptors, OPNs play a role in modulating ion channels, particularly in the process of phototransduction. In response to light, OPNs trigger changes in the ionic permeability of the membrane, leading to hyperpolarization or depolarization of the cell.
- Activation of Downstream Signaling Pathways: OPNs activation is also associated with the activation of downstream signaling pathways that are specific to the type of OPN and the cell type where it is expressed. These pathways can include the activation of protein kinases, transcription factors, and, ultimately, changes in gene expression.

These pathways collectively contribute to the cellular response to light, encompassing visual signaling in the retina and optosensing in the skin. Additionally, they are involved in various non-visual light responses in other tissues and organisms to such an extent that, in relatively recent times, OPNs have been defined as “polymodal sensory receptors” [26]. In the context of phototransduction, these proteins are classified based on their amino acid sequences and the specific wavelengths of light they are sensitive to. Each OPN is typically assigned a specific number or name based on its characteristics. In Vertebrates retina, two primary types of OPNs are present in visual cells. As anticipated, cones are responsible for color vision and visual acuity in bright light conditions. They contain various types of OPN1 (or cone opsin or photopsin) that are sensitive to different wavelengths of light, contributing to color vision. Three types of OPN1 are present: short-wavelength (S) OPN1, with the highest sensitivity to blue light (around 420–440 nm); medium-wavelength (M) OPN1, which is the most responsive to green light (around 534–545 nm); and long-wavelength (L) OPN1, which is particularly sensitive to red light (around 564–580 nm). The combined signals from these three types of OPN1s allow humans to perceive a broad spectrum of colors and achieve visual acuity (or sharpness), which is the ability of the eye to perceive and sharply define details in bright light conditions. Rods contain OPN2 or rhodopsin, which is highly sensitive to low light levels. This sensitivity enables us to see in dimly lit environments, as it exhibits an exceptional sensitivity to light, capable of detecting even a single photon. OPN3, also referred to as encephalopsin or panopsin, is a light-sensitive pigment found in the retina of the eye and other tissues such as the skin, brain, and adipose tissue. Together with melanopsin (OPN4), it is involved in the regulation of various physiological processes, including the sleep–wake cycle, mood, and hormonal balance. OPN3 exhibits heightened sensitivity to blue light, which is abundant in natural sunlight as well as electronic devices like smartphones and computers. Additionally, OPN3 is implicated in the formation of the supranuclear melanin cap that shields keratinocytes from the harmful effects of the exposure to UVA radiation [27,28]. Although OPN3 is highly expressed in different brain areas, its precise function in these regions remains unidentified, aside from modulating the Acoustic Startle Reflex (ASR), which is an involuntary, rapid contraction of skeletal muscle in response to a sudden, intense auditory stimulus [29]. Notably, in adipocytes, OPN3 has been linked to the conversion of white adipose tissue to thermogenic brown adipose tissue [30], suggesting a thermosensitive nature for OPN3. This could explain its presence in body areas not typically exposed to light rays, such as adipose tissue and even the heart, as described in an interesting article by Prof. de Lauro Castrucci’s group [7]. As anticipated, OPN4 (or melanopsin) is a photopigment found in specialized cells of the retina and in the skin. Melanopsin responds to different wavelengths of light, including blue light and longer wavelengths associated with both daylight and twilight. As anticipated, it is primarily expressed in ipRGCs, a specialized type of photoreceptor cell serving as an additional pathway for light detection, playing a crucial role in photoentrainment and in the regulation of physiological responses to changes in light intensity. In addition to the retina, OPN4 expression has also been detected in other non-ocular tissues and cells, including the skin, where it is expressed in melanocytes. Here, melanopsin is thought to be involved in the regulation of various light-mediated processes, such as melanin production and skin pigmentation, potentially contributing to non-visual, light-induced effects on skin biology and physiology [31,32]. Finally, OPN5 is a photopigment mainly found in the retina and brain. This is why it is also known as neuropsin [33]. Interestingly, it contributes to the light-dependent development of blood vessels in the retina and is essential for engaging the local circadian rhythm in both the retina and cornea. Additionally, during corneal wounding after an injury, it is upregulated and becomes light-sensitive [34,35,36]. Recently, Lan and colleagues have demonstrated that OPN5 is a key player in epidermal melanogenesis [37]. They described that its activation by UV radiation is followed by signal transduction through Ca^2+^-dependent GPCRs and protein kinase C pathways. This process contributes to the regulation of microphthalmia-associated transcription factor (MITF), which serves as the key regulator of the melanocyte lineage and certain pigmentation genes, thereby facilitating downstream cellular effects in melanocytes.

## 5. Effects of Skin Exposition to UV Radiation

As anticipated, throughout life, human skin is exposed to sunlight, whose ultraviolet component has the highest potential to damage its structural and physiological integrity. It has long been understood that to counteract the effects of this exposure, a continuum of cellular strategies operates in our skin. As described by Rastogi and colleagues [38], initial molecular damage induced by ultraviolet rays includes the formation of covalent bonds between adjacent pyrimidine bases in DNA, which are the origin of thymine dimers and CPDs. These photoproducts distort the normal structure of the DNA helix and can interfere with its proper functioning. UV radiation, particularly UVB and UVA, facilitates the isomerization of trans-urocanic acid to its cis-isoform. Urocanic acid is a naturally occurring compound found in the stratum corneum, the outermost layer of the skin. Its isomerization is significant because the cis urocanic acid form is known to have immunosuppressive properties, as it stimulates regulatory T cells (Treg) [12], potentially making the skin more susceptible to the harmful effects of UV radiation. UV radiation can also stimulate the production and release of phospholipids, including platelet-activating factor (PAF). These molecules contribute to the modulation of immune and inflammatory pathways and influence the skin’s defense mechanisms to UV-induced damage [39]. Additionally, UV exposure triggers the activity of catalase, nitric oxide synthase and the generation of ROS. This process, recently described by Vieyra-Garcia and Wolf [13], activates the inflammasome and suppresses immune responses within skin cells, representing a peculiar characteristic of the skin’s reaction to environmental stress.

## 6. Light on the AhR/FICZ Connection

Already in the 80s, the Rannug group demonstrated that in the skin exposed to solar radiation, the essential amino acid Trp absorbs UV radiation, generating various photoproducts, including 6-formylindolo[3,2-b] carbazole (FICZ) [40]. FICZ is a potent endogenous ligand for Aryl hydrocarbon Receptor (AhR), an evolutionary conserved transcription factor playing a role in diverse cellular processes and biological responses. Remarkably, this endogenous metabolite has a binding affinity to AhR that exceeds that of the exogenous 2,3,7,8-tetrachlorodibenzo-p-dioxin (TCDD), a compound known as having the highest affinity for the AhR binding site.

AhR is a widely expressed protein that, as anticipated, functions as a promiscuous ligand-activated transcription factor. It is known for its role in xenobiotic metabolism, particularly in the detoxification of harmful substances. It plays a crucial role in the response to environmental toxins, such as polycyclic aromatic hydrocarbons (PAHs), polychlorinated biphenyls (PCBs), dioxins, and other pollutants. Besides its well-known role in sensing environmental toxins, it binds to a plethora of endogenous ligands and is involved in various physiological processes, including immune responses, inflammation, cell growth, and proliferation. AhR belongs to the basic helix-loop-helix (bHLH)/PAS protein family, but, unlike other members of this group, AhR is not embedded in membranes and resides in the cytoplasm. Here, AhR is inactivated by a multi-molecular complex consisting of (1) two 90 kDa Heat Shock Proteins (HSP90s) that maintain AhR in a ligand-binding-favorable conformation [41]; (2) one AhR-Interacting Protein (AIP, also denominated XAP2 or Ara9), which stabilizes the interaction between AhR and HSP90 [42]; (3) one p23 protein, which counteracts AhR ubiquitination and degradation [43], and, last but not least, (4) the signaling partner protein tyrosine kinase pp60(c-Src). This phosphoprotein is a member of the Src family of tyrosine kinases, which play a crucial role in regulating protein dynamics at cell–matrix interfaces and have important functions in cellular growth control [44]. Pp60(c-Src) is the most abundant tyrosine kinase in fibroblasts. It is involved in various cellular processes, including cancer development, as the DNA segment encoding for the Src protein is classified as a proto-oncogene that, when activated, is directly involved in tumorigenesis. Intriguingly, this enzyme is the instigator of an interesting crosstalk between AhR and several pathways dependent on growth factors.

In 1999, the Bock group demonstrated that in liver cancer cells, after TCDD binding, pp60Src detaches from the AhR inactivating complex and migrates to the membrane. Here, it interacts with the Epithelial Growth Factor Receptor (EGFR) and activates the MAPK cascade. This, in turn, stimulates the transcription of Matrix Metalloproteinase-1 (MMP-1) genes and Cyclooxygenase -2 (COX-2), the isoform of the cyclooxygenase family associated with pain and inflammation [45]. Later, the Ueda group evidenced the activation of EGFR and the induction of COX-2 expression upon the short-term exposure of human skin cells to UVB irradiation [46]. They also considered that earlier studies demonstrated that the selective inhibition of COX-2 could reduce the formation of skin tumors caused by photo carcinogenesis in mice [47]. On these bases, they hypothesized that COX-2 might play a significant role in the development of skin tumors caused by UV exposure. Keeping in mind that EGFR can also be activated by oxidative stress [48], the Ueda group demonstrated that oxidative stress, along with the activation of EGFR, ERK, p38 MAP kinase, and PI3-kinase, are important factors in the UVB-induced expression of COX-2. Interestingly, they cited c-Src activation as a step following keratinocytes exposition to UV, but they did not link it to EGFR activation, as previously described by the Weber and Parsons groups [49,50]. These latter authors in the 1990s gave evidence that the proteins pp60Src produced by the oncogene v-Src and its cellular counterpart c-Src have a strong ability to phosphorylate and activate EGFR, thus providing evidence about the plausible role of pp60(c-v-Src) in tumorigenesis and mitogenesis. Both groups performed their studies in fibroblasts cell lines, where, since 1983, the presence of AhR had already been reported by the Dufresne group [51]. In the thin red line linking the FICZ-mediated activation of AhR to the EGFR-mediated expression of COX2, the connecting role of c-Src had not yet been considered.

**Figure 1:**
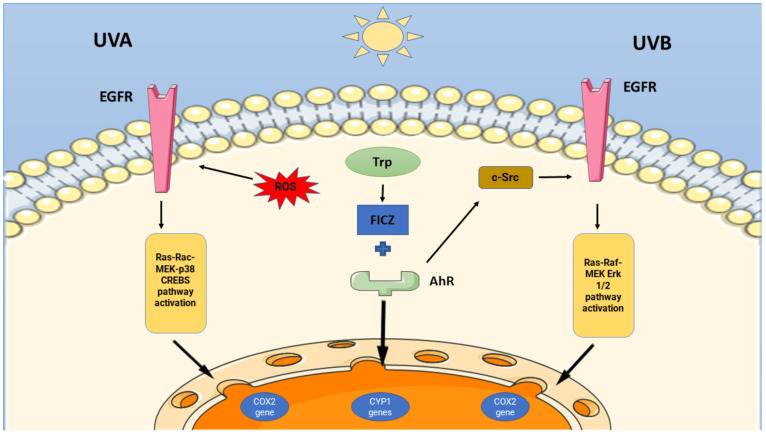
The effects of UVA and UVB radiation on skin tryptophan lead to pro-inflammatory pathways. Following UVB exposure (right side), Trp is converted into FICZ, which binds to AhR. The binding-associated conformational modification leads to the dissociation of c-Src from the AhR inactivating complex. This tyrosine-kinase protein phosphorylates EGFR, whose activation initiates the downstream Ras-Raf-MEK-Erk 1/2 signaling pathway, thus leading to the transcriptional activation of the COX-2 gene. Activated AhR translocates to the nucleus, where it promotes the transcription of CYP1 genes. Exposure to UVA radiation (right side) sensitizes FICZ and promotes the generation of Reactive Oxygen Species (ROS), which activates EGFR in a ligand-independent feature. This triggers the downstream Ras-Rac-MEKs-p38 pathway, culminating in CREB (cAMP regulatory element binding protein) activation and translocation to the nucleus, where it triggers the transcriptional activation of the COX-2 gene. The figure was partly generated using Servier Medical Art, provided by Servier (https://www.servier.com) (Suresnes, France), licensed under a Creative Commons Attribution-NoDerivatives 4.0 International. The website (https://smart.servier.com) was accessed on 29 April 2024 [61].

As previously shown by Rannug’s group [40], in 2007, the Krutmann group observed that exposure to UVB in the immortalized human keratinocytes HaCaT cell line stimulated the formation of FICZ, which behaves as a chromophore and photosensitizer [52]. They provided evidence that, following its binding to AhR, the chaperone c-Src is released and triggers EGFR activation. Consistent with the findings of the Ueda group in 2003 [46], they recognized a key role for EGFR, as this receptor, after c-Src-mediated activation, can interact with other kinases and signaling molecules as part of its signaling cascade. These include (1) the MAP kinase ERK 1–2, which stimulates the transcription of COX2, the key enzyme in the production of prostaglandins and in generating inflammatory conditions [53], and (2) p38, a pro-apoptotic class of MAP kinases responsive to stress stimuli such as cytokines, ultraviolet irradiation, heat, and osmotic shocks [54]. Inizio moduloThese mechanisms, which they described only in UV-exposed wild type mice but not in AhR KO mice, suggested a pivotal role for AhR in both nuclear and cytoplasmic pathways occurring after UVB exposition. Moreover, they provided clear evidence of AhR’s involvement as an active participant in certain inflammatory pathways following UV exposure, as confirmed later by other researchers [55,56]. Interestingly, the Ueda group demonstrated the shuttling of the activated AhR to the nucleus, where it influences the transcription of the xenobiotic-metabolizing enzyme CYP1A1 gene (Figure 1). After an initial inhibition, FICZ is the most efficient inducer of CYP1A1 gene expression and a good substrate for the CyP1A1 enzyme, which participates in its metabolism, in a negative feedback auto-regulatory pathway [57]. This point provides a logical explanation for Youssef’s data, showing a low concentration of FICZ in UVB-exposed human keratinocytes and in their culture medium [58]. This feedback system plays a vital role in FICZ-activated AHR signaling, as it regulates the equilibrium between the quiescence and proliferation of various cell types, including intra-thymic progenitor cells, as well as hematopoietic, pulmonary, and neuro-epithelial stem cells [59]. In addition to CYP1A1, the Mukhtar group demonstrated in 2000 the induction of CYP1B1 in the human epidermis by UVB light [60], thus providing evidence of a possible role for both CyP1A1 and CyP1B1 in UV-induced skin cancers, as both activate pro-carcinogenic compounds and convert them into carcinogenic metabolites.

### 6.1. Beyond the Light: Focus on the FICZ Metabolic Role

**Figure 2:**
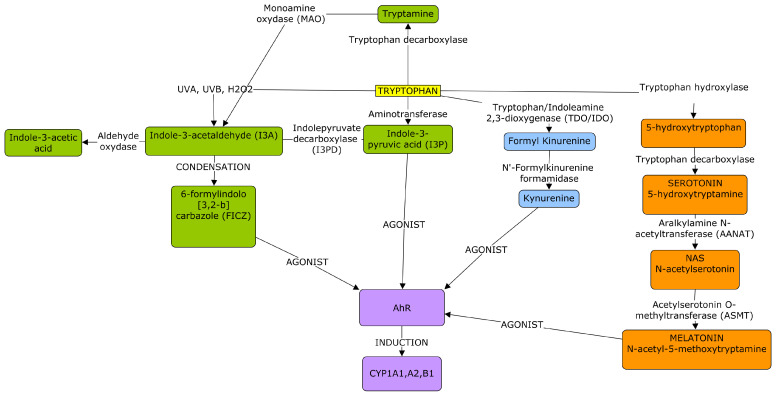
Tryptophan metabolites interact with AhR and potentially interfere with serotonin and melatonin synthesis. The essential amino acid tryptophan (Trp) can be transformed by different metabolic pathways. From left to right, it can be decarboxylated by the enzyme tryptophan decarboxylase, generating Tryptamine, which is oxidated by the monoamine oxidase (MAO) or by hydrogen peroxide (H_2_O_2_)/UVA/UVB to Indole-3-acetaldehyde (I3A). From Trp, an aminotransferase generates Indole-3-pyruvic acid (I3P), which is transformed by Indolepyruvate decarboxylase (I3PD) in Indole-3-acetaldehyde (I3A). Then, the condensation of I3A generates 6-formylindolo[3,2-b]carbazole (FICZ), the best endogenous agonist of AhR. The right side of the figure shows the reaction that, starting from Trp, is catalyzed by the two enzymes tryptophan/Indoleamine 2,3-dioxygenase (TDO/IDO). The product is Formyl Kynurenine, the substrate transformed by the enzyme N’-Formylkinurenine formamidase in Kynurenine, another endogenous agonist of AhR. Finally, the hydroxylation of Trp by tryptophan hydroxylase generates 5-hydroxytryptophan, which is decarboxylated by tryptophan decarboxylase in 5-hydroxytryptamine, aka Serotonin. The aralkylamine N-acetyltransferase (AANAT) transforms Serotonin in N-acetyl serotonin (NAS), which is finally transformed by Acetylserotonin O-methyltransferase (ASMT) in N-acetyl-5-methoxytryptamine, aka Melatonin. Melatonin activates AhR, which induces CYP1A1, while FICZ-activated AhR induces the expression of CYP1A1/A2/B1. This map was drawn using the freeware software CmapTools, version 6.04, developed by the Florida Institute for Human and Machine Cognition (IHMC) (Cmap|CmapTools (https://ihmc.us), accessed on 29 April 2024.

In 2009, the Rannug group demonstrated the high affinity of FICZ to CyP1B1, CyP1A2, sulfotransferases (SULTs) 1A1, 1A2, 1B1, and 1E1. They also identified some sulfo-conjugated compounds of FICZ in human urine, highlighting the need to clarify the origin of FICZ, whether it is photolytic, metabolic, or nutritional [62]. In 2016, the same group showed compelling evidence for a systemic origin of FICZ, with indole-3-acetaldehyde (I3A) as a crucial precursor resulting from the enzymatic deamination of tryptamine by the enzyme Monoamine Oxidase (MAO) and/or the decarboxylation of indole-3-pyruvate (I3P) by the enzymes indole-3-pyruvate decarboxylase (I3PD) (Figure 2). Interestingly, I3A can also be the result of the oxidation of Trp by Hydrogen Peroxide (H_2_O_2_). Then, its condensation gives rise to FICZ. This pathway is less important than the direct photolytic pathway for FICZ formation in the skin, but it is prevalent for FICZ formation in inner tissues, such as the liver and lungs. These reactions suggest a light-independent origin of FICZ [63]. This point is of pivotal importance, as it also attributes a metabolic role to FICZ, providing a molecular basis that explains how light can affect complex cellular and systemic pathways. This issue was successfully addressed in a compelling paper by the Baglole group. Their research findings demonstrated that exposing mice’s skin to a minimal erythemic dose of UVB results in a change in AhR signaling in various peripheral organs, thereby impacting immune system regulation [64]. Following an in vitro protocol mimicking in vivo 1–2 minimal erythemal doses, they observed the activation of AhR and its translocation to the nucleus in human epithelial-like, leukemia monocytic, and keratinocytes cell lines. Within a few minutes, they detected the induction of some target genes: CYP1A1, CCL1 (C-C Motif Chemokine Ligand 1, a chemotactic factor expressed by activated T cells), S100A9 (S100 Calcium Binding Protein A9, a cytoplasmic and/or nuclear protein regulating the cell cycle and differentiation), and IL (interleukin) 10, IL22, and IL23. In later in vivo experiments, they noticed that even the serum from mice exposed to moderate UVB irradiation activated some AHR target genes, such as CYP1A1 in ex vivo fibroblasts and the Treg markers IL17a and IL17b in naïve T cells from treated mice. These data suggest the release of one or more AhR agonists into the serum following UVB treatment; more importantly, they highlight that UV exposure of the skin triggers AhR-related signaling not only in the skin itself but also in peripheral tissues and, in particular, in the immune system. In this framework, many authors had already focused their attention on the tryptophan photoproduct FICZ. In 2015, Park and colleagues [8] showed that FICZ, independent of AhR binding, has a potent photosensitizing effect at nanomolar concentrations, enhancing UVA-induced oxidative stress and DNA damage in keratinocytes, both in vivo and in vitro. Based on this, they defined FICZ as the “most potent endogenous UVA photosensitizer identified till date”. As effectively summarized by Syed and Mukhtar [65], FICZ is a per se photosensitizer which, in addition to visible light, is able to absorb both UVA and UVB radiation. In the first case, FICZ mediates and potentiates UVA phototoxic effects, resulting in the death of keratinocyte cells in reconstructed epidermal tissue and murine skin (Figure 1).

The photosensitivity induced by FICZ is linked to the generation of intracellular oxidative stress and oxidative DNA damage, without the involvement of AhR. In parallel, UVB exposure stimulates the formation of FICZ, which binds and activates AhR. Consequently, AhR releases Src, leading to the activation of EGFR and downstream inflammatory signaling, which significantly bolsters the UVB stress response. As suggested by Wincent and colleagues [66,67], this negative feedback can be turned off by a plethora of exogenous molecules that bind AhR, causing a progressive rise in the FICZ concentration, thus interfering with its participation to inflammation [68,69], UV responses [66], and circadian rhythms [70].

### 6.2. FICZ/AhR Effects on The Circadian Rhythms

As recently explained by Tischkau and colleagues [70], AhR is involved in circadian clock regulation. Light exposure, besides inducing the photooxidative formation of FICZ, leads to heightened glutamatergic neurotransmission. This results in an increase in intracellular calcium levels, MAPK activation, the phosphorylation of CREB, and the CRE-mediated transcription of the period circadian regulator 1 (Per1) gene, a leading player of circadian rhythms regulation. If AhR is activated, it decreases the rhythm’s amplitude, which, on the contrary, increases if AhR is inhibited. This is due to different strategies: activated AhR can associate with BMAL1 (Brain and muscle ARNT-like protein-1). This heterodimer can replace the CLK (Clock, another circadian regulator gene): BMAL1 complex, which usually binds to EBox elements in the promoter of Per1. Inizio moduloAs a consequence, the transcription of light-activated Per1 decreases. In addition, AhR can activate JNK, which halts the interaction of the CREB:CBP (CREB binding protein) complex with the Cre elements in the Per1 promoter; again, as a result, the expression of light-induced Per1 decreases. Intriguingly, the CLK:BMAL1 complex regulates the transcription of AhR through a direct interaction with EBox elements in its promoter. In turn, this interaction is inhibited by PER 1 and PER2. In navigating this intricate web of feedback connections, it is crucial to remember that FICZ stands out as the most effective, high-affinity, light-induced endogenous AhR activator. Being generated for the most part by the photooxidation of Trp, it links light exposure to the regulation of circadian rhythms through the active participation of AhR. Examining the pathways involving Trp, it becomes evident that its photosensing intersects and potentially interferes with circadian rhythms.

As is well known, since light is a powerful inhibitor of melatonin synthesis, it can worsen the quality of sleep and modify the sleep–wake cycle. This interference is particularly evident in stress-related inflammation if it triggers the activation of Indoleamine 2,3-dioxygenase (IDO-1), an enzyme converting Trp into Kyn, and tryptophan 2,3-dioxygenase (TDO), which produces kynurenic acid (KynA, 4-Hydroxyquinoline-2-carboxylic acid) [71,72]. Some authors have suggested a close interplay between these tryptophan catabolite (TRYCAT) pathways and the melatonergic pathway, as TRYCAT diverts Trp from serotonin production, an essential precursor of melatonin (Figure 2). Moreover, Kyn and KynA bind and activate AhR, triggering the expression of CyP1s, which, together with other factors, regulates the N-acetyl-serotonin (NAS)/melatonin ratio [73]. Interestingly, the photosensitive melatonin, whose secretion peaks from dusk onwards, can inhibit the activity of AhR and vice versa. As a matter of fact, melatonin induces the isoform alpha 7 of the nicotinic acetylcholine receptor (nAChR α7), which is involved in some psychiatric disorders. Interestingly, nAChR α7 is inactivated by TRYCAT. On the other side, TRYCAT activates AhR, which, as already described, induces CyP1s and melatonin metabolism [74].

However, the precise nature of these interactions and their physiological implications are not yet completely clear and require further research to be fully understood. Hence, during inflammatory conditions, melatonin levels are reduced, together with anti-inflammatory and antioxidant properties. So, inflammatory conditions that involve TRYCAT-mediated activation can inhibit serotonin and melatonin synthesis during the night as well, thus affecting their balance and potentially giving rise to sleep and mood disturbances [75]. In this framework, however, one should also consider the balance with the anti-inflammatory effects of FICZ, which, if generated by photooxidation, can be exerted only during daylight [76].

### 6.3. FICZ: A Possible Player in Skin Photodynamic Therapy?

As anticipated, FICZ formation in the skin is predominantly linked to the photooxidation of Trp, which, both in vitro and in vivo, is an efficient UVA/Visible light photosensitizer, with absorbance maximum at 390 nm. In 2022, the Wondrak group assayed its photodynamic properties in a set of carcinogenic and melanoma cell lines exposed to UVA irradiation. Here, they described their apoptotic cell death due to intense oxidative stress, suggesting the possible use of FICZ for the in vivo photooxidative elimination of malignant cells, a promising therapeutical strategy intensely investigated in the last decade [8,77]. These data were based on previous studies in a murine model, showing that UVA treatment promotes the generation of ROS through the induction of CYP1s [78]. ROS stimulate a strong oxidation of both proteins and nuclear DNA. They also decrease the enzymatic activity of the NER (nucleotide excision repair) pathway, which eliminates large DNA adducts resulting from UV irradiation and exposure to chemical mutagens. These phenomena determine intense cellular stress, followed by the expression of typical markers such as HSP70 and caspase 3, leading up apoptotic cell death [8]. In this context, the exposure to UVA is crucial, serving as a key factor in maintaining the continuous production of FICZ. FICZ undergoes rapid metabolism by AhR-induced CYP1s [58,65], and its activity would decrease if not for the ongoing UVA-induced photooxidation of Trp, ensuring its constant supply. As already described, in cells exposed to both UVA and UVB rays, FICZ triggers inflammation either by oxidative stress or by the pp60Src tyrosine kinase/EGFR/MAP Kinase/COX2 pathway. This framework has been further investigated by the Furue group, who detailed the involvement of the FICZ-AhR-ROS pathway in upregulating the expression of the proinflammatory cytokines IL1A, IL1B, and IL6 in UVB-irradiated HaCaT keratinocytes. [79]. The Furue group also observed that the interaction between FICZ and AhR resulted in an increased expression of filaggrin, loricrin, and involucrin. These proteins are part of the terminal differentiation program of keratinocytes, a process essential for epidermal differentiation. Within this framework, the FICZ-AhR complex also activates OVOL-1, a transcription factor strongly linked to epithelial differentiation and necessary for the upregulation of filaggrin and loricrin, but not involucrin.

Inizio moduloThese findings provide a conceptual foundation for understanding the beneficial effects of controlled sun exposure in conditions like atopic dermatitis (AD) and other skin disorders characterized by impaired skin barrier function. Moreover, it is plausible to speculate that the topical application of FICZ could be advantageous in such cases [80]. As an example, FICZ shows promise as a potential therapeutic agent for treating scleroderma. This chronic dermatologic disease, due to abnormalities in TNF signaling, mainly involves connective tissue, where collagen over-production and deposition occurs. Interestingly, the Mitoma group demonstrated that in normal human dermal fibroblasts, FICZ activates matrix metalloproteinase 1 (MMP1) through the AHR/MEK/ERK signaling pathway, thus exerting a possible anti-fibrotic effect [81]. Furthermore, irrespective of AhR interaction, FICZ proficiently inhibits the Transforming Growth Factor beta (TGF-β)-induced elevation of both mRNA and protein levels of α2-smooth muscle actin and collagen I, along with actin polymerization in myofibroblasts. Thus, it contributes to the anti-fibrotic effects induced by UV [82].

### 6.4. FICZ/AhR Modulation of the Immune System

In the last decade, thanks to the Rannug group’s research, FICZ has been strongly related to the modulation of immune system activity [59,83,84]. In epithelial barriers, the integument, and the mucous membranes, FICZ is generated not only through UV-light exposure but also through enzyme-catalyzed processes in microorganisms or dietary intake. Here, it undergoes a rapid metabolism and elimination following the AhR-mediated induction of the CyP1A1 enzyme [58]. In the presence of CyP1A1 inhibitors hindering FICZ clearance, it can trigger the IL-22 pathway. IL-22 belongs to the IL-10 cytokine family and, depending on the context, can display both proinflammatory and tissue-protective functions. Within the skin, IL-22 regulates immune responses by binding to IL-22R, which is its receptor in epithelial cells. This interaction stimulates the secretion of tissue-specific cytokines, chemokines, and antimicrobial agents, which recruit neutrophils. FICZ, whether generated through host metabolism from tryptophan or administered systemically (e.g., via intraperitoneal injection), induces the AhR-mediated production of IL-22 in various immune cells.

Thus, regardless of whether FICZ is endogenously produced or originates from an external source, it plays a functional role in maintaining or restoring immune homeostasis [59,85]. A similar framework, involving the small quantity of FICZ derived from microbiota metabolism, has been described by Agneta Rannug in the intestinal barrier [83]. In a recent study, the Bieber group demonstrated that FICZ-activated AhR in epidermal Langerhans cells maintains the expression of the immunosuppressive enzyme IDO-1 and downregulates FcεRI, the high-affinity immunoglobulin E receptor. Notably, FcεRI expression is high in the Langerhans cells of people with atopic dermatitis (AD). This anti-inflammatory effect provides a theoretical explanation for why UVB phototherapy reduces immune cell infiltration, particularly cells expressing FcεRI, in skin lesions of patients with AD [86]. It is now clear that the participation of AhR in immune functions includes the regulation of the expression of cytokines such as IL-10, IL-21, and IL-22 and the modulation of the differentiation and the balance between Treg and Th17 cells [87]. Within this context, the Layseca-Espinosa group demonstrated the involvement of FICZ in modulating the adaptive immune response. Considering the role of dendritic cells (DCs) as antigen-presenting cells which express AhR, they investigated the potential impact of AhR activation on immune tolerance using an in vitro model. Their research revealed that human DCs, when exposed to FICZ during their maturation process, upregulate the expression of the immunosuppressive enzyme IDO-1 while concurrently downregulating the expression of IL6 and TNF-α. Additionally, they observed that FICZ-treated DCs promote the differentiation of naïve T lymphocytes into T regulatory (Treg)-like cells expressing CD4^+^, CD25high, and Foxp3. These Treg cells effectively inhibit the activation of autologous T lymphocytes [88]. Similar results were also obtained by other laboratories, confirming that AhR ligands affect the differentiation of T cells; in particular, the response to the administration of FICZ is dose- and time-dependent. In 2018, an in vivo study by the Kerkvliet group [89] showed that low doses of FICZ led to temporary CYP1A1 expression, without triggering the generation of Tregs or suppressing the alloresponse. Instead, the production of the pro-inflammatory cytokine IL-17 increased. At higher doses, FICZ administration suppressed the alloresponse in a time course, initially fostering the emergence of the anti-inflammatory Foxp3-Tr1 cells and subsequently amplifying the population of natural immunosuppressive Foxp3^+^ Tregs cells. Compared to the immunosuppressive role of TCDD, occurring even at low doses and in early stages, low doses of FICZ, which is rapidly metabolized by CyP1A1, are proinflammatory in early stages, while high doses elicit an immunosuppressive effect. Considering that keratinocytes exhibit an approximately twofold induction in CYP1A1 [58], this could explain the therapeutic effect of high doses of FICZ when administered in an in vitro model of psoriasis [76]. Hence, alongside FICZ’s anti-fibrotic properties, we may consider an immunomodulatory mechanism that could explain its advantageous effects in specific skin pathology models.

### 6.5. FICZ and Blue Light

FICZ has also been described as a sensitizer to blue light, a part of the visible light spectrum with both potential benefits and drawbacks. Being the part with the lower wavelength, blue light has a high energy content, which is useful in the skin photodynamic therapy of some pathologies, as in facial actinic keratosis. In this skin condition, the exposure of the photosensitizer protoporphyrin IX to blue light induces the generation of ROS and immunogenic cell death (ICD), a form of regulated cell death that triggers an immune response against the dying cells [90]. This generates an inflammatory response that helps to reduce the precancerous lesions, which are a typical hallmark of this pathology. In 2007, Denda and colleagues observed the reduction in the lipid layer between the stratum corneum and the granular layer in hairless mice whose skin barrier had been disrupted and exposed to blue light. So, in this case, blue light delays the recovery of the skin barrier, unlike what happens after exposure to red light [91]. Recently, in a reconstructed human skin model, the Duplan group showed that exposure to blue light impairs the repair mechanism of pyrimidine dimers, a typical kind of UVB-induced DNA damage [92]. In this complex and contradictory framework, this band of electromagnetic radiation is currently under scrutiny, due to the widespread and prolonged exposure of human skin and eyes to blue light-emitting devices, such as computers and smartphones. [66].

Interestingly, in 2022, Hammond and coworkers described AhR expression in parts of the eye and in different cell types of the retina [93]. They showed that FICZ activates AhR, which has a protective effect against blue light exposure, as observed in AhR^-^/AhR^-^mice, where blue light can damage the retinal pigmented epithelial cell, belonging to the blood–retina barrier [94]. Consistently, in human keratinocytes, Becker and colleagues [95] showed that, after 30 min of blue light irradiation (432 nm), phase I and II metabolism genes belonging to the “AhR gene battery” were upregulated, while cell cycle and apoptosis genes were downregulated. Considering the high content of Trp in keratinocytes, these data support the hypothesis of AhR activation by some products of tryptophan photooxidation, particularly the endogenous FICZ, which could mediate this effect of photobiomodulation. In this case, AhR is supposed to exert a cell-protective effect, characterized by reduced oxidative stress, cell proliferation and inflammatory responses, coupled with an increased production of steroid hormones that reinforce the anti-inflammatory action. Delving into these mechanisms could pave the way for novel therapeutic strategies offering better protection against blue light emission.

## 7. Conclusions and Perspectives

As outlined in this paper, electromagnetic radiation, particularly UV and visible light, interacts with photosensitive molecules within the skin, triggering various pathways that result in a range of biological responses. While some of these responses may have protective roles, such as melanogenesis, their main purpose is to connect the body and its essential functions to the alternating day and night external environment. Essentially, they synchronize our physiology with this rhythm, thereby supporting and regulating crucial processes such as sleep–wake cycles, hormone production, and immune function. This connection is facilitated by certain photosensitive molecules that can act as chromophores, photosensitizers, or both, as in the case of FICZ. Our interest in this molecule stems from the fact that, aside from its metabolic origin, its formation mainly comes from the photoconversion of Trp. This process is prevalent in UV-exposed epidermis due to its high Trp content. In this light-driven communication between the skin and internal systems, FICZ gains importance due to its strong binding affinity with AhR, a widely recognized cellular transducer of both external and internal signals. We have described how the FICZ/AhR complex influences some functional networks in both healthy and pathological conditions. We have examined their interactions in metabolism, immune system function, and the regulation of circadian rhythms, and we propose the FICZ/AhR complex as a potential mediator of the effects of UV and visible light on human skin. Expanding on this idea, we suggest AhR as a transductor that relays the metabolic signals of certain light-responsive molecules. Hence, we finally propose AhR as a promising contributor to skin optotransduction and a potential target for therapeutic interventions. In our view, this research area holds intriguing implications for developing effective therapeutic strategies.

## Figures and Tables

**Table 1:** List of some optosensitive molecules, with their anatomical location, physiological role, activating stimuli, range of wavelength sensitivity, and peak absorbance.

Denomination	Abbreviation	Localization	Physiologic Role	Stimulus	Wavebands
Melanin [7]		Melanocytes Skin appendages Substantia nigra locus coeruleus	Pigmentation Thermoregulation Antioxidant Neuroprotection	UV, visible, infrared	300–800 nm
Photopsin Cone Opsin [7]	OPN1	Cones in the retina	Color vision		380–780 nm
Short-wavelength Opsin 1	(S)OPN1	Cones in the retina	Color vision	blue light	420–440 nm
Medium-wavelenght Opsin 1	(M)OPN1	Cones in the retina	Color vision	green light	534–545 nm
Long- wavelenght Opsin 1	(L)OPN1	Cones in the retina	Color vision	red light	564–580 nm
Rhodopsin Opsin 2 [7]	OPN2	Rods in the retina	Black and white vision	low levels visible light	Peak at 498 nm (visible light)
Encephalopsin Panopsin [7]	OPN3	Retina, skin, brain, adipose tissue.	sleep–wake cycle skin pigmentation acoustic startle reflex thermogenic adipose tissue	blue to green spectral range	Peak at 445 nm (blue/green light)
Melanopsin [7]	OPN4	Retinal ipRGCs, melanocytes	melanin production skin pigmentation photoentrainment	UVA, blue light	Peak at 480 nm (blue light)
Neuropsin [7]	OPN5	Retinal GCs, cornea, melanocytes	pupillary light reflex visual sensitivity circadian rhythms mood-related behaviors	UV	Peak at 380 nm (UVA)
6-Formylindolo[3,2-B] Carbazole [8]	FICZ	Epithelial, endothelial, immune, neuronal cells, adipocytes, hepatocytes	responses to environmental stimuli regulation of gene expression immune modulation regulation of circadian rhythms skin homeostasis	UVB, UVA	Peak at 390 nm (UVA-visible light)
Flavin Adenine Dinucleotide and reduced forms [7,9,10]	FAD, FADH, FADH2	Mitochondria and cytoplasm	changes in cellular signaling regulation of circadian rhythms photorepair of DNA damage	UV, Blue	340–450 nm
Nicotinamide Adenine Dinucleotide, Nicotinamide Adenine Dinucleotide Phosphate, and reduced forms [7,10]	NAD, NADH, NADH2 NADP, NADPH, NADPH2	Mitochondria and cytoplasm	electron carriers in redox reactions cofactors in light-responsive metabolic pathways signaling molecules in photosensing pathways regeneration of antioxidants light sensing and signal transduction	UVB, UVA	240–400 nm
Carotenoids [7,10]		plant and animal pigments	tissue pigmentation light harvesting and energy transfer processes non-photochemical quenching (dissipation of energy excess) light signalling pathways	UVB, UVA, Visible light	250–550 nm
Heme Group [11]		blood vessels in muscle, skin, retina	Oxygenation of tissues involved in optosensing processes	Blue–green visible light	400–600 nm
Lipofuscin [7,9,10]		lysosomes	Scavenger of ROS generated by exposure to light	UV–visible light	300−500 nm
Urocanic Acid [7,10,11,12]		skin	Photoprotection against UVB		200–320 nm

## References

[1] Wong R., Geyer S., Weninger W., Guimberteau J.C., Wong J.K. (2016). The dynamic anatomy and patterning of skin. Exp. Dermatol..

[2] Marzvanyan A., Alhawaj A.F. (2024). Physiology, Sensory Receptors. [Updated 14 August 2023]. StatPearls.

[3] Neville J.J., Palmieri T., Young A.R. (2021). Physical Determinants of Vitamin D Photosynthesis: A Review. JBMR Plus.

[4] Duda M., Domagalik A., Orlowska-Feuer P., Krzysztynska-Kuleta O., Beldzik E., Smyk M.K., Stachurska A., Oginska H., Jeczmien-Lazur J.S., Fafrowicz M. (2020). Melanopsin: From a small molecule to brain functions. Neurosci. Biobehav. Rev..

[5] Kusumoto J., Takeo M., Hashikawa K., Komori T., Tsuji T., Terashi H., Sakakibara S. (2020). OPN4 belongs to the photosensitive system of the human skin. Genes Cells.

[6] de Assis L.V.M., Lacerda J.T., Moraes M.N., Domínguez-Amorocho O.A., Kinker G.S., Mendes D., Silva M.M., Menck C.F.M., Câmara N.O.S., de Lauro Castrucci A.M. (2022). Melanopsin (Opn4) is an oncogene in cutaneous melanoma. Commun. Biol..

[7] Monteiro de Assis L.V., Newton Tonolli P., Moraes M.N., Baptista M.S., de Lauro Castrucci A.M. (2021). How does the skin sense sunlight? An integrative view of light sensing molecules. J. Photochem. Photobiol. C Photochem. Rev..

[8] Park S.L., Justiniano R., Williams J.D., Cabello C.M., Qiao S., Wondrak G.T. (2015). The Tryptophan-Derived Endogenous Aryl Hydrocarbon Receptor Ligand 6-Formylindolo[3,2-b]Carbazole Is a Nanomolar UVA Photosensitizer in Epidermal Keratinocytes. J. Investig. Dermatol..

[9] Bäumler W., Regensburger J., Knak A., Felgenträger A., Maisch T. (2012). UVA and endogenous photosensitizers--the detection of singlet oxygen by its luminescence. Photochem. Photobiol. Sci..

[10] Baier J., Maisch T., Maier M., Engel E., Landthaler M., Bäumler W. (2006). Singlet oxygen generation by UVA light exposure of endogenous photosensitizers. Biophys. J..

[11] Barresi C., Stremnitzer C., Mlitz V., Kezic S., Kammeyer A., Ghannadan M., Posa-Markaryan K., Selden C., Tschachler E., Eckhart L. (2011). Increased sensitivity of histidinemic mice to UVB radiation suggests a crucial role of endogenous urocanic acid in photoprotection. J. Investig. Dermatol..

[12] Schwartz T. (2005). Mechanisms of UV-induced immunosuppression. Keio J. Med..

[13] Vieyra-Garcia P.A., Wolf P. (2018). From Early Immunomodulatory Triggers to Immunosuppressive Outcome: Therapeutic Implications of the Complex Interplay Between the Wavebands of Sunlight and the Skin. Front. Med..

[14] Pavan W.J., Sturm R.A. (2019). The Genetics of Human Skin and Hair Pigmentation. Annu. Rev. Genom. Hum. Genet..

[15] Fu C., Chen J., Lu J., Yi L., Tong X., Kang L., Pei S., Ouyang Y., Jiang L., Ding Y. (2020). Roles of inflammation factors in melanogenesis. Mol. Med. Rep..

[16] Hossain M.R., Ansary T.M., Komine M., Ohtsuki M. (2021). Diversified Stimuli-Induced Inflammatory Pathways Cause Skin Pigmentation. Int. J. Mol. Sci..

[17] Liu Y., Liang Y., Zhou X., Dent J.E., di Nardo L., Jiang T., Qin D., Lu Y., He D., Nardini C., Podbielska H., Kapalla M. (2023). Wound Healing from Bench to Bedside: A PPPM Bridge Between Physical Therapies and Chronic Inflammation. Predictive, Preventive, and Personalised Medicine: From Bench to Bedside. Advances in Predictive, Preventive and Personalised Medicine.

[18] Young A.R. (1997). Chromophores in human skin. Phys. Med. Biol..

[19] de Freitas L.F., Hamblin M.R. (2016). Proposed Mechanisms of Photobiomodulation or Low-Level Light Therapy. IEEE J. Sel. Top. Quantum Electron..

[20] Leyane T.S., Jere S.W., Houreld N.N. (2021). Cellular Signalling and Photobiomodulation in Chronic Wound Repair. Int. J. Mol. Sci..

[21] Wondrak G.T., Jacobson M.K., Jacobson E.L. (2006). Endogenous UVA-photosensitizers: Mediators of skin photodamage and novel targets for skin photoprotection. Photochem. Photobiol. Sci..

[22] Correia J.H., Rodrigues J.A., Pimenta S., Dong T., Yang Z. (2021). Photodynamic Therapy Review: Principles, Photosensitizers, Applications, and Future Directions. Pharmaceutics.

[23] de Melo Gomes L.C., de Oliveira Cunha A.B., Peixoto L.F.F., Zanon R.G., Botelho F.V., Silva M.J.B., Pinto-Fochi M.E., Góes R.M., de Paoli F., Ribeiro D.L. (2023). Photodynamic therapy reduces cell viability, migration, and triggers necroptosis in prostate tumor cells. Photochem. Photobiol. Sci..

[24] Liebert A., Pang V., Bicknell B., McLachlan C., Mitrofanis J., Kiat H. (2022). A Perspective on the Potential of Opsins as an Integral Mechanism of Photobiomodulation: It’s Not Just the Eyes. Photobiomodul. Photomed. Laser Surg..

[25] Gether U. (2000). Uncovering molecular mechanisms involved in activation of G protein-coupled receptors. Endocr. Rev..

[26] Leung N.Y., Montell C. (2017). Unconventional Roles of Opsins. Annu. Rev. Cell Dev. Biol..

[27] Lan Y., Zeng W., Wang Y., Dong X., Shen X., Gu Y., Zhang W., Lu H. (2023). Opsin 3 mediates UVA-induced keratinocyte supranuclear melanin cap formation. Commun. Biol..

[28] Regazzetti C., Sormani L., Debayle D., Bernerd F., Tulic M.K., De Donatis G.M., Chignon-Sicard B., Rocchi S., Passeron T. (2018). Melanocytes Sense Blue Light and Regulate Pigmentation through Opsin-3. J Invest. Dermatol..

[29] Upton B.A., Nayak G., Schweinzger I., D’Souza S.P., Vorhees C.V., Williams M.T., Earl B.R., Lang R.A. (2022). Comprehensive Behavioral Analysis of Opsin 3 (Encephalopsin)-Deficient Mice Identifies Role in Modulation of Acoustic Startle Reflex. eNeuro.

[30] Ekechukwu O.N., Christian M. (2022). Metabolic responses of light and taste receptors—Unexpected actions of GPCRs in adipocytes. Rev. Endocr. Metab. Disord..

[31] Semo M., Peirson S., Lupi D., Lucas R.J., Jeffery G., Foster R.G. (2003). Melanopsin retinal ganglion cells and the maintenance of circadian and pupillary responses to light in aged rodless/coneless (rd/rd cl) mice. Eur. J. Neurosci..

[32] Kelley J.L., Davies W.I.L. (2016). The Biological Mechanisms and Behavioral Functions of Opsin-Based Light Detection by the Skin. Front. Ecol. Evol. Sect. Behav. Evolut. Ecol..

[33] Tarttelin E.E., Bellingham J., Hankins M.W., Foster R.G., Lucas R.J. (2003). Neuropsin (Opn5): A novel opsin identified in mammalian neural tissue. FEBS Lett..

[34] Nguyen M.T., Vemaraju S., Nayak G., Odaka Y., Buhr E.D., Alonzo N., Tran U., Batie M., Upton B.A., Darvas M. (2019). An opsin 5-dopamine pathway mediates light-dependent vascular development in the eye. Nat. Cell Biol..

[35] Van Gelder R.N., Buhr E.D. (2016). Ocular Photoreception for Circadian Rhythm Entrainment in Mammals. Annu. Rev. Vis. Sci..

[36] Díaz N.M., Lang R.A., Van Gelder R.N., Buhr E.D. (2020). Wounding Induces Facultative Opn5-Dependent Circadian Photoreception in the Murine Cornea. Investig. Ophthalmol. Vis. Sci..

[37] Lan Y., Zeng W., Dong X., Lu H. (2021). Opsin 5 is a key regulator of ultraviolet radiation-induced melanogenesis in human epidermal melanocytes. Br. J. Dermatol..

[38] Rastogi R.P., Richa K.A., Tyagi M.B., Sinha R.P. (2010). Molecular mechanisms of ultraviolet radiation-induced DNA damage and repair. J. Nucleic Acids.

[39] Tse B.C.Y., Ferguson A.L., Koay Y.C., Grau G.E., Don A.S., Byrne S.N. (2023). Exposure to solar ultraviolet radiation establishes a novel immune suppressive lipidome in skin-draining lymph nodes. Front. Immunol..

[40] Rannug A., Rannug U., Rosenkranz H.S., Winqvist L., Westerholm R., Agurell E., Grafström A.K. (1987). Certain photooxidized derivatives of tryptophan bind with very high affinity to the Ah receptor and are likely to be endogenous signal substances. J. Biol. Chem..

[41] Antonsson C., Whitelaw M.L., McGuire J., Gustafsson J.A., Poellinger L. (1995). Distinct roles of the molecular chaperone hsp90 in modulating dioxin receptor function via the basic helix-loop-helix and PAS domains. Mol. Cell. Biol..

[42] Meyer B.K., Perdew G.H. (1999). Characterization of the AhR-hsp90-XAP2 core complex and the role of the immunophilin-related protein XAP2 in AhR stabilization. Biochemistry.

[43] Petrulis J.R., Perdew G.H. (2002). The role of chaperone proteins in the aryl hydrocarbon receptor core complex. Chem. Biol. Interact..

[44] Enan E., Matsumura F. (1996). Identification of c-Src as the integral component of the cytosolic Ah receptor complex, transducing the signal of 2,3,7,8-tetrachlorodibenzo-p-dioxin (TCDD) through the protein phosphorylation pathway. Biochem. Pharmacol..

[45] Köhle C., Gschaidmeier H., Lauth D., Topell S., Zitzer H., Bock K.W. (1999). 2,3,7,8-Tetrachlorodibenzo-p-dioxin (TCDD)-mediated membrane translocation of c-Src protein kinase in liver WB-F344 cells. Arch. Toxicol..

[46] Ashida M., Bito T., Budiyanto A., Ichihashi M., Ueda M. (2003). Involvement of EGF receptor activation in the induction of cyclooxygenase-2 in HaCaT keratinocytes after UVB. Exp. Dermatol..

[47] Pentland A.P., Schoggins J.W., Scott G.A., Khan K.N., Han R. (1999). Reduction of UV-induced skin tumors in hairless mice by selective COX-2 inhibition. Carcinogenesis.

[48] Peus D., Vasa R.A., Meves A., Pott M., Beyerle A., Squillace K., Pittelkow M.R. (1998). H2O2 is an important mediator of UVB-induced EGF-receptor phosphorylation in cultured keratinocytes. J. Investig. Dermatol..

[49] Wasilenko W.J., Payne D.M., Fitzgerald D.L., Weber M.J. (1991). Phosphorylation and activation of epidermal growth factor receptors in cells transformed by the src oncogene. Mol. Cell. Biol..

[50] Belsches A.P., Haskell M.D., Parsons S.J. (1997). Role of c-Src tyrosine kinase in EGF-induced mitogenesis. Front. Biosci..

[51] Okey A.B., Mason M.E., Gehly E.B., Heidelberger C., Muncan J., Dufresne M.J. (1983). Defective binding of 3-methylcholanthrene to the Ah receptor within C3H/1OT1/2 clone 8 mouse fibroblasts in culture. Eur. J. Biochem..

[52] Fritsche E., Schäfer C., Calles C., Bernsmann T., Bernshausen T., Wurm M., Hübenthal U., Cline J.E., Hajimiragha H., Schroeder P. (2007). Lightening up the UV response by identification of the aryl hydrocarbon receptor as a cytoplasmatic target for ultraviolet B radiation. Proc. Natl. Acad. Sci. USA.

[53] Guarnieri T. (2016). Non-Steroidal Anti-Inflammatory Drugs As Gatekeepers Of Colon Carcinoma Highlight New Scenarios Beyond Cyclooxygenases Inhibition. Curr. Cancer Drug Targets.

[54] Han J., Wu J., Silke J. (2020). An overview of mammalian p38 mitogen-activated protein kinases, central regulators of cell stress and receptor signaling. F1000Research.

[55] Vogeley C., Esser C., Tüting T., Krutmann J., Haarmann-Stemmann T. (2019). Role of the Aryl Hydrocarbon Receptor in Environmentally Induced Skin Aging and Skin Carcinogenesis. Int. J. Mol. Sci..

[56] Noakes R. (2015). The Aryl Hydrocarbon Receptor: A Review of Its Role in the Physiology and Pathology of the Integument and Its Relationship to the Tryptophan Metabolism. Int. J. Tryptophan Res..

[57] Ma Q. (2011). Influence of light on aryl hydrocarbon receptor signaling and consequences in drug metabolism, physiology and disease. Expert. Opin. Drug Metab. Toxicol..

[58] Youssef A., von Koschembahr A., Caillat S., Corre S., Galibert M.D., Douki T. (2019). 6-Formylindolo[3,2-b]carbazole (FICZ) is a Very Minor Photoproduct of Tryptophan at Biologically Relevant Doses of UVB and Simulated Sunlight. Photochem. Photobiol..

[59] Rannug A., Rannug U. (2018). The tryptophan derivative 6-formylindolo[3,2-b]carbazole, FICZ, a dynamic mediator of endogenous aryl hydrocarbon receptor signaling, balances cell growth and differentiation. Crit. Rev. Toxicol..

[60] Katiyar S.K., Matsui M.S., Mukhtar H. (2000). Ultraviolet-B exposure of human skin induces cytochromes P450 1A1 and 1B1. J. Investig. Dermatol..

[61] Servier Medical Art. https://smart.servier.com/.

[62] Wincent E., Amini N., Luecke S., Glatt H., Bergman J., Crescenzi C., Rannug A., Rannug U. (2009). The suggested physiologic aryl hydrocarbon receptor activator and cytochrome P4501 substrate 6-formylindolo[3,2-b]carbazole is present in humans. J. Biol. Chem..

[63] Smirnova A., Wincent E., Vikström Bergander L., Alsberg T., Bergman J., Rannug A., Rannug U. (2016). Evidence for New Light-Independent Pathways for Generation of the Endogenous Aryl Hydrocarbon Receptor Agonist FICZ. Chem. Res. Toxicol..

[64] Memari B., Nguyen-Yamamoto L., Salehi-Tabar R., Zago M., Fritz J.H., Baglole C.J., Goltzman D., White J.H. (2019). Endocrine aryl hydrocarbon receptor signaling is induced by moderate cutaneous exposure to ultraviolet light. Sci. Rep..

[65] Syed D.N., Mukhtar H. (2015). FICZ: A Messenger of Light in Human Skin. J. Investig. Dermatol..

[66] Wincent E., Bengtsson J., Mohammadi Bardbori A., Alsberg T., Luecke S., Rannug U., Rannug A. (2012). Inhibition of cytochrome *P*4501-dependent clearance of the endogenous agonist FICZ as a mechanism for activation of the aryl hydrocarbon receptor. Proc. Natl. Acad. Sci. USA.

[67] Wincent E., Kubota A., Timme-Laragy A., Jönsson M.E., Hahn M.E., Stegeman J.J. (2016). Biological effects of 6-formylindolo[3,2-b]carbazole (FICZ) in vivo are enhanced by loss of CYP1A function in an AhR2-dependent manner. Biochem. Pharmacol..

[68] Guarnieri T., Abruzzo P.M., Bolotta A. (2020). More than a cell biosensor: Aryl hydrocarbon receptor at the intersection of physiology and inflammation. Am. J. Physiol. Cell Physiol..

[69] Bock K.W. (2020). Aryl hydrocarbon receptor (AHR)-mediated inflammation and resolution: Non-genomic and genomic signaling. Biochem. Pharmacol..

[70] Tischkau S.A. (2020). Mechanisms of circadian clock interactions with aryl hydrocarbon receptor signalling. Eur. J. Neurosci..

[71] Guarnieri T. (2022). Hypothesis: Emerging Roles for Aryl Hydrocarbon Receptor in Orchestrating CoV-2-Related Inflammation. Cells.

[72] Anderson G., Reiter R.J. (2020). Melatonin: Roles in influenza, Covid-19, and other viral infections. Rev. Med. Virol..

[73] Mazzoccoli G., Kvetnoy I., Mironova E., Yablonskiy P., Sokolovich E., Krylova J., Carbone A., Anderson G., Polyakova V. (2021). The melatonergic pathway and its interactions in modulating respiratory system disorders. Biomed. Pharmacother..

[74] Anderson G., Maes M. (2017). Interactions of Tryptophan and Its Catabolites with Melatonin and the Alpha 7 Nicotinic Receptor in Central Nervous System and Psychiatric Disorders: Role of the Aryl Hydrocarbon Receptor and Direct Mitochondria Regulation. Int. J. Tryptophan Res..

[75] Anderson G., Jacob A., Bellivier F., Geoffroy P.A. (2016). Bipolar Disorder: The Role of the Kynurenine and Melatonergic Pathways. Curr. Pharm. Des..

[76] Di Meglio P., Duarte J.H., Ahlfors H., Owens N.D., Li Y., Villanova F., Tosi I., Hirota K., Nestle F.O., Mrowietz U. (2014). Activation of the aryl hydrocarbon receptor dampens the severity of inflammatory skin conditions. Immunity.

[77] Lin Y., Zhen L., Wei X., Kai J., Jinliang L., Xiaohui Z., Yong Z., Yihan W. (2023). Towards overcoming obstacles of type II photodynamic therapy: Endogenous production of light, photosensitizer, and oxygen. Acta Pharm. Sin. B.

[78] Hrycay E.G., Bandiera S.M. (2015). Involvement of Cytochrome P450 in Reactive Oxygen Species Formation and Cancer. Adv. Pharmacol..

[79] Tanaka Y., Uchi H., Hashimoto-Hachiya A., Furue M. (2018). Tryptophan Photoproduct FICZ Upregulates IL1A, IL1B, and IL6 Expression via Oxidative Stress in Keratinocytes. Oxid. Med. Cell. Longev..

[80] Furue M., Uchi H., Mitoma C., Hashimoto-Hachiya A., Tanaka Y., Ito T., Tsuji G. (2019). Implications of tryptophan photoproduct FICZ in oxidative stress and terminal differentiation of keratinocytes. G. Ital. Dermatol. Venereol..

[81] Murai M., Yamamura K., Hashimoto-Hachiya A., Tsuji G., Furue M., Mitoma C. (2018). Tryptophan photo-product FICZ upregulates AHR/MEK/ERK-mediated MMP1 expression: Implications in anti-fibrotic phototherapy. J. Dermatol. Sci..

[82] Murai M., Tsuji G., Hashimoto-Hachiya A., Kawakami Y., Furue M., Mitoma C. (2018). An endogenous tryptophan photo-product, FICZ, is potentially involved in photo-aging by reducing TGF-β-regulated collagen homeostasis. J. Dermatol. Sci..

[83] Rannug A. (2020). How the AHR Became Important in Intestinal Homeostasis-A Diurnal FICZ/AHR/CYP1A1 Feedback Controls Both Immunity and Immunopathology. Int. J. Mol. Sci..

[84] Rannug A. (2022). 6-Formylindolo[3,2-b]carbazole, a Potent Ligand for the Aryl Hydrocarbon Receptor Produced Both Endogenously and by Microorganisms, can Either Promote or Restrain Inflammatory Responses. Front. Toxicol..

[85] Sonnenberg G.F., Fouser L.A., Artis D. (2009). Functional Biology of the IL-22-IL-22R Pathway in Regulating Immunity and Inflammation at Barrier Surfaces. Adv. Immunol..

[86] Koch S., Stroisch T.J., Vorac J., Herrmann N., Leib N., Schnautz S., Kirins H., Förster I., Weighardt H., Bieber T. (2017). AhR mediates an anti-inflammatory feedback mechanism in human Langerhans cells involving FcεRI and IDO. Allergy.

[87] Quintana F.J., Sherr D.H. (2013). Aryl hydrocarbon receptor control of adaptive immunity. Pharmacol. Rev..

[88] Jurado-Manzano B.B., Zavala-Reyes D., Turrubiartes-Martínez E.A., Portales-Pérez D.P., González-Amaro R., Layseca-Espinosa E. (2017). FICZ generates human tDCs that induce CD4^+^ CD25^high^ Foxp3^+^ Treg-like cell differentiation. Immunol. Lett..

[89] Ehrlich A.K., Pennington J.M., Bisson W.H., Kolluri S.K., Kerkvliet N.I. (2018). TCDD, FICZ, and Other High Affinity AhR Ligands Dose-Dependently Determine the Fate of CD4+ T Cell Differentiation. Toxicol. Sci..

[90] Shen A.S., Heusinkveld L.E., Updyke A., Nowacki A.S., Warren C.B., Maytin E.V. (2024). Painless photodynamic therapy for facial actinic keratoses: A retrospective cohort study of the post-treatment inflammatory response. Photodiagn. Photodyn. Ther..

[91] Denda M., Fuziwara S. (2008). Visible radiation affects epidermal permeability barrier recovery: Selective effects of red and blue light. J. Investig. Dermatol..

[92] Douki T., Bacqueville D., Jacques C., Geniès C., Roullet N., Bessou-Touya S., Duplan H. (2024). Blue light impairs the repair of UVB-induced pyrimidine dimers in a human skin model. Photochem. Photobiol..

[93] Hammond C.L., Roztocil E., Gupta V., Feldon S.E., Woeller C.F. (2022). More than Meets the Eye: The Aryl Hydrocarbon Receptor is an Environmental Sensor, Physiological Regulator and a Therapeutic Target in Ocular Disease. Front. Toxicol..

[94] Kim S.Y., Yang H.J., Chang Y.S., Kim J.W., Brooks M., Chew E.Y., Wong W.T., Fariss R.N., Rachel R.A., Cogliati T. (2014). Deletion of aryl hydrocarbon receptor AHR in mice leads to subretinal accumulation of microglia and RPE atrophy. Investig. Ophthalmol. Vis. Sci..

[95] Becker A., Klapczynski A., Kuch N., Arpino F., Simon-Keller K., De La Torre C., Sticht C., van Abeelen F.A., Oversluizen G., Gretz N. (2016). Gene expression profiling reveals aryl hydrocarbon receptor as a possible target for photobiomodulation when using blue light. Sci. Rep..

